# Efficacy of *Trichoderma* spp. spore suspensions against cotton-infesting spider mites (Acari: Tetranychidae)

**DOI:** 10.1007/s10493-026-01137-8

**Published:** 2026-05-01

**Authors:** İsmail Asrav, Yunus Korkom, Alireza Saboori, Ibrahim Cakmak

**Affiliations:** 1 Graduate School of Natural and Applied Sciences, Aydin Adnan Menderes University, Aydin, Turkey; 2 Department of Plant Protection, Faculty of Agriculture, Aydin Adnan Menderes University, Aydin, Turkey; 3 Jalal Afshar Zoological Museum, Department of Plant Protection, Faculty of Agriculture, University of Tehran, Karaj, Iran

**Keywords:** Cotton, *Tetranychus urticae*, *Tetranychus turkestani*, *Trichoderma afroharzianum*, Biological control, Integrated pest management

## Abstract

Spider mites, particularly *Tetranychus urticae* Koch and *T. turkestani* Ugarov and Nikolskii (Acari: Tetranychidae), are major agricultural pests, and their increasing resistance to chemical acaricides underscores the need for alternative control strategies. This study explored the biocontrol potential of *Trichoderma* spp. against these mites. Specifically, we evaluated (1) the efficacy of spore suspensions from five isolates of four *Trichoderma* species (*Trichoderma afroharzianum*, *T. guizhouense*, *T. harzianum* and *T. virens*) and their combinations on different biological stages of *T. urticae*, (2) the efficacy of *T. afroharzianum* spore suspension on different populations of *T. urticae* (green and red forms) and *T. turkestani* in both Petri dish and pot experiments. Results showed that *Trichoderma* spore suspensions were ineffective against *T. urticae* eggs, with mortality rates ≤ 2.8% at 7 days post-application (dpa), which did not differ significantly from the control. However, the fungi caused significant mortality of 50–65% in the mobile stages of the mites (larvae, nymphs, and adult females) compared with the control. Based on the initial screening results, a single *Trichoderma* isolate (*T. afroharzianum*) was selected for detailed assessment across multiple spider mite populations. Petri dish assays at 7 dpa showed that *T. afroharzianum* was more effective against *T. turkestani* (up to 60%) and the green form of *T. urticae* (53%) than against the red form of *T. urticae* (35%). Pot experiments confirmed these results, showing that *T. afroharzianum* reduced egg and mobile stage populations by 36–39% in *T. turkestani* and *T. urticae* (green form and laboratory culture populations), whereas reductions in the red form of *T. urticae* were not statistically significant. These findings suggest that *T. afroharzianum* has potential as a biological control agent; however, its efficacy varies among spider mite populations, highlighting the need to integrate it with other biological or chemical strategies.

## Introduction

Cotton (*Gossypium hirsutum* L.) is an important fiber crop worldwide, serving as a fundamental raw material for the textile and clothing industries and a source of cottonseed oil. This oil is not only widely used in the food industry but has gained traction as a renewable feedstock for biodiesel production, thereby reducing dependence on fossil fuels and their associated environmental impacts. Consequently, cotton plays a crucial role not only in the textile sector but also in energy agriculture (Basal et al. 2019).

Globally, cotton cultivation is widespread, with Asia leading the way at approximately 63% of the world’s cotton production, followed by the Americas (20%) and Africa (14%). A small number of countries dominate the market, with India, China, the United States, Brazil, Pakistan, Türkiye, and Uzbekistan collectively producing 84% of the world’s supply. Türkiye ranks eleventh in both total production and imports, and is also a major consumer (Özüdoğru 2021). Within the country, the Aegean Region, particularly the provinces of Aydin, Izmir, and Denizli, accounts for nearly 30% of national cotton production (Günaydin et al. 2019).

Despite its economic importance, cotton production is significantly threatened by various biotic factors as diseases, weeds and, particularly in Aydin province, spider mites. These pests, primarily *Tetranychus urticae* (red and green forms) and *Tetranychus turkestani* (Acari: Tetranychidae), colonize the underside of cotton leaves, causing chlorotic spots that lead to reddening, drying, and eventual leaf drop. Infestations before squaring delay crop development, while post-squaring attacks cause abscission of floral buds, flowers, and bolls, significantly reducing yield (Sulek and Cakmak 2022; Sulek et al. 2023; Yüksel et al. 2024). Spider mites also produce dense webs, which facilitate aerial dispersal and rapid spread. Current control primarily relies on chemical acaricides; however, frequent applications have led to environmental concerns and widespread resistance. For example, Duran et al. (2024) reported that *T. urticae* red form populations in Aydin have exhibited high resistance against common acaricides, including spiromesifen (982–1246 fold), etoxazole (410–517 fold), abamectin (187–224 fold), and hexythiazox (156–168 fold). In contrast, *T. turkestani* populations remain mostly susceptible, though some resistance to hexythiazox (164–182 fold) was noted. This high resistance, coupled with the mites’ rapid reproduction, highlights the urgent need for alternative control strategies.

Biological control offers the most promising alternatives for pest control. The predatory mites *Phytoseiulus persimilis*, *Neoseiulus californicus* and *Amblyseius swirskii* (Acari: Phytoseiidae) are widely used against spider mites in greenhouses (Cakmak et al. 2005, 2009; Yalçın et al. 2023), and their use in open-field crops is growing. Additionally, entomopathogenic fungi (*Beauveria*, *Metarhizium*, *Lecanicillium*), as well as bacterial secondary metabolites (*Xenorhabdus* and *Photorhabdus)* have demonstrated efficacy in suppressing spider mites (Dogan et al. 2017; Eroglu et al. 2019; Cevizci et al. 2020; Incedayi et al. 2021).

Recent research has expanded the role of *Trichoderma* species (Hypocreaceae: Ascomycota), traditionally known as mycoparasites and plant growth–promoting fungi, to include acaricidal activity against spider mites. Multiple studies have demonstrated that exposing different developmental stages of *T. urticae* to *Trichoderma* spore suspensions or metabolites results in significant mortality (Afifi et al. 2007; Sholla and Kottb 2017; Elhakim et al. 2020; Meteab et al. 2022; Awad and Hendawy 2023; Çobanoğlu et al. 2024; Evren et al. 2025). Reported effects vary widely, achieving 25% to over 80% mortality depending on the specific *Trichoderma* species, formulation, temperature, and mite life stage. These findings suggest that *Trichoderma* spp. could serve as promising biocontrol agents against spider mites in cotton agroecosystems.

This study investigated the efficacy of spore suspensions from five isolates of four local *Trichoderma* species (*T. afroharzianum*,* T. guizhouense*,* T. harzianum* and *T. virens*) obtained from soils in Aydin province on cotton spider mites. The main objectives were: (i) to evaluate the effects of spore suspensions of different *Trichoderma* species, alone and in combination, on different developmental stages of *T. urticae*, (ii) to assess the efficacy of *T. afroharzianum* spore suspensions against different populations of *T. urticae* (red and green forms) and *T. turkestani* in both Petri dish and pot experiments.

## Materials and methods

### Plant growing

Bean plants (*Phaseolus vulgaris* var. barbunia) were cultivated for the maintenance of separately reared populations of *Tetranychus urticae* (green and red forms) and *T. turkestani*, and for use in experimental studies. Seeds were sown in 15 cm diameter plastic pots filled with a peat-perlite mixture in a 3:1 ratio. Pots were irrigated regularly with tap water. Plants were grown in controlled-environment climate chambers maintained at 25 ± 1 °C and 60 ± 10% relative humidity (RH) under a 16:8 h light: dark photoperiod. No fertilization was applied during plant cultivation.

### Collection, identification and rearing of *Tetranychus urticae* and *Tetranychus turkestani*

#### Field collection

*Tetranychus urticae* (green and red forms) and *T. turkestani* populations were collected from cotton-growing districts (Germencik, Kocarli, Nazilli and Soke) in Aydin province during July-August 2023. Cotton leaves presenting typical spider mite symptoms (chlorotic spots, leaf bronzing, or webbing) were sampled directly from the field. Samples were placed into sealed paper bags, then into plastic zipper bags to reduce moisture loss, and immediately transferred into insulated ice boxes and transported to the Acarology Laboratory of Aydin Adnan Menderes University. Samples were stored at + 4 °C until examination.

#### Laboratory examination and identification

Leaves were examined under a stereomicroscope (Leica EZ4, Leica Microsystems GmbH, Wetzlar, Germany), and mites were identified using both morphological and molecular techniques.

#### Morphological identification

For taxonomic confirmation, at least 10 permanent slides were prepared from both males and females of each field population. Specimens were mounted in Hoyer’s medium (females dorsally, males dorsoventrally) and cured in a drying oven (Memmert UN110, Memmert GmbH & Co. KG, Schwabach, Germany) at 50 °C for 5 days. Identification was performed under a light microscope (Zeiss Axio Imager A2, Carl Zeiss, Germany) using standard taxonomic keys, based primarily on the shape of the aedeagus in males and the dorsal, ventral, and leg setae patterns in females, which are key features for distinguishing *Tetranychus* species (Pritchard and Baker 1955; Seeman and Beard 2011; Yüksel et al. 2024).

#### Molecular identification

For DNA-based confirmation, one adult female from each population was individually placed into a sterile 1.5 mL microcentrifuge tube under a stereomicroscope. DNA extraction and species identification were performed following the protocol of Yüksel et al. (2024), using the Internal Transcribed Spacers (ITS) and Cytochrome Oxidase I mitochondrial gene (COI) regions for species-level differentiation.

#### Establishment of laboratory colonies

A total of nine populations were obtained, consisting of three populations each of green *T. urticae*, red *T. urticae*, and *T. turkestani* (Table 1). These populations were reared simultaneously in two formats: Petri dishes and containers. In the Petri dish, large Petri dishes (15 cm diameter) were lined with moist cotton wool to maintain leaf turgidity. A detached bean leaf (abaxial side upward) was placed on the cotton, and one mated female from each population was transferred with a fine brush. At least two Petri dishes per population were prepared and placed in separate water-filled trays (6.5 L) with Vaseline-coated rim to prevent mite escape and cross-contamination. Leaves were replaced when senescent, and dishes were moistened three times a week. For container cultures, each population was maintained in a separate 22.5 × 33.5 × 30.5 cm container with ventilation holes. A potted bean plant was placed inside, and mites were transferred from the Petri dish cultures with a fine brush. All populations were kept in a climate-controlled room at 25 ± 1 °C and 60 ± 10% RH, and a 16:8 h light: dark photoperiod.

**Table 1:** Origin of *Tetranychus urticae* (green and red forms) and *T. turkestani* populations collected from cotton fields in Aydin province, Türkiye

Species	Collection date	District	Code	Coordinates	
*Tetranychus urticae* green form	12.07.2023	Nazilli	G1	37°51’35”N	28°17’46”E
	10.07.2023	Soke	G2	37°43’4”N	27°24’51”E
	07.07.2023	Germencik	G3	37°50’56”N	27°37’2”E
*Tetranychus urticae* red form	10.07.2023	Soke	R1	37°43’4”N	27°24’51”E
	02.08.2023	Germencik	R2	37°47’46”N	27°30’51”E
	24.07.2023	Kocarli	R3	37°44’53”N	27°48’4”E
*Tetranychus turkestani*	12.07.2023	Nazilli	1	37°50’45”N	28°20’6”E
	10.07.2023	Soke	2	37°38’24”N	27°24’32”E
	24.07.2023	Kocarli	3	37°45’10”N	27°47’13”E

### Fungal isolates and preparation of *Trichoderma* spore suspensions

The study utilized five *Trichoderma* isolates obtained from different field soils in Aydin in a previous study (Korkom 2022) and identified through molecular methods: *T. afroharzianum* Tr132 (GenBank: OP847792.1), *T. guizhouense* Tr49 (GenBank: OP703665.1), *T. guizhouense* Tr118 (GenBank: OP703666.1), *T. harzianum* Tr124 (GenBank: OP825136.1) and *T. virens* Tvr2 (GenBank: MZ853844.1). Molecular identification details are available in GenBank under the corresponding accession numbers. These fungal isolates were cultured on potato dextrose agar (PDA) at 25 °C for 7 days under a 12 h light/12 h dark photoperiod in an incubator (Memmert IF55, Germany). To prepare the spore suspensions, 0.05% (v/v) Tween 20 in sterile distilled water was added to the surface of cultures. Spores were dislodged using a sterile glass rod, vortexed for homogenization, and filtered through four layers of sterile gauze to remove mycelial fragments (Al-Hazmi and TariqJaveed 2016; Korkom 2022). Spore concentration was determined with a hemocytometer under a light microscope (Olympus CX21, Japan) and adjusted to 1 × 10^8^ spores/mL for each isolate.

### Effects of *Trichoderma* spore suspensions on different biological stages of *Tetranychus urticae*

A laboratory-maintained culture population of *T. urticae* (green form, originally collected from Germencik cotton fields in 2017) was used and reared as described above. To ensure developmental synchrony, all developmental stages were obtained from synchronized cohorts established by allowing adult females to oviposit on bean leaves for 24 h, after which adults were removed. Eggs laid within this period developed under controlled conditions, and individuals of the same age were used for each developmental stage (egg, larva, protonymph, deutonymph, and adult female). The susceptibility of different developmental stages to *Trichoderma* isolates was then evaluated. Petri dishes (9 cm diameter) were lined with cotton wool moistened with distilled water, and a detached bean leaf (5.5 cm diameter) was placed abaxial side upward on the cotton. Using a fine-tipped brush under a stereomicroscope, 20 individuals of each stage were transferred onto each leaf. Treatments consisted of five isolates (*Trichoderma afroharzianum* Tr132, *T. guizhouense* Tr49, *T. guizhouense* Tr118, *T. harzianum* Tr124, and *T. virens* Tvr2) and their combinations, prepared by mixing spore suspensions of the respective isolates at a 1:1 (equal volume) ratio (*Trichoderma guizhouense* Tr49 + Tr118; *T. guizhouense* Tr118 + *T. harzianum*; *T. afroharzianum* + *T. harzianum*; *T. virens* + *T. harzianum*; *T. afroharzianum* + *T. virens*), totaling 10 treatments. Spore suspensions were applied using a custom-built spray tower (Burkard Manufacturing, UK) at 1 atm pressure, delivering 2 mL per Petri dish. The control was sprayed with sterile distilled water containing 0.05% (v/v) Tween 20. Mortality was recorded at 5 and 7 days post-application (dpa) under a stereomicroscope; these time points were selected based on previous studies (Dogan et al. 2017; Evren et al. 2025) to capture both early and intermediate mortality responses. Egg viability was assessed based on chorion condition, with nonviable eggs exhibiting a crumpled chorion without larval emergence, whereas viable eggs showed larval emergence or visible exit holes in the chorion. For mobile stages, individuals were considered dead if they did not respond to gentle prodding with a fine-tipped brush. Dead mites were collected separately for each treatment. Mite cadavers were placed in Petri dishes containing a *Trichoderma* selective medium supplemented with chloramphenicol to confirm the presence of *Trichoderma* within the mite bodies (Rodríguez-González et al. 2022). Each treatment was conducted with five replicates per biological stage, and the experiment was repeated four times. The study was carried out in climate chambers at 25 ± 1 °C, 60 ± 10% RH, and 16 h light.

### Effects of spore suspension of *Trichoderma afroharzianum* on different spider mite species and populations

#### Petri dish experiments

*Trichoderma afroharzianum* Tr132, selected from the previous experiment (Effects of *Trichoderma* spore suspensions on different biological stages of *T. urticae*), was selected as a representative isolate for further testing on nine populations (three populations from each species or form) of *T. urticae* (green and red forms) and *T. turkestani* obtained from cotton fields in Germencik, Kocarli, Nazilli and Soke districts. The experimental setup was identical to the first experiment, except that only adult females were tested. Adult females used in the experiments were obtained from synchronized laboratory cultures of uniform age. Each treatment consisted of 20 adult females transferred to bean leaves, sprayed with 2 mL spore suspension of *T. afroharzianum* using a spray tower (1 atm). Controls were treated with sterile distilled water containing 0.05% (v/v) Tween 20. Mortality was recorded at 5 and 7 dpa. Individuals that showed no movement upon gentle prodding with a fine-tipped brush were classified as dead. Each treatment was conducted with five replicates per population and repeated four times in climate chambers at 25 ± 1 °C, 60 ± 10% RH, and a 16-h photoperiod.

#### Pot experiments

The effect of *T. afroharzianum* spore suspension on four populations of *T. urticae* (green and red forms and culture) and *T. turkestani* was also tested in pot experiments. Bean plants were grown in 7 × 5 cm pots until the two-cotyledon stage. To standardize plant size, one cotyledon was removed, leaving a single leaf per pot (Incedayi et al. 2021; Evren et al. 2025). For each spider mite population, a total of 60 individuals (10 per stage: egg, larva, protonymph, deutonymph, adult female and male) were transferred to the plants under a stereomicroscope using a fine brush. All developmental stages used in the pot experiments were obtained from synchronized laboratory-reared colonies to ensure uniform age structure. Plants were then sprayed with 5 mL of *T. afroharzianum* suspension on both leaf surfaces using a hand sprayer. The same volume of sterile distilled water containing 0.05% (v/v) Tween 20 was sprayed on plants in the control group. Seven days after treatment, leaves were removed, and all living mites present on both the upper and lower leaf surfaces were counted separately by developmental stage under a stereomicroscope. Each treatment consisted of five replicates and was repeated four times in climate chambers at 25 ± 1 °C, 60 ± 10% RH, and a 16-h photoperiod.

### Statistical analysis

Data from the first experiment were analyzed using the Generalized Linear Model (GLM), and mean comparisons were performed with Tukey’s HSD test. In the second experiment, mortality rates from the petri dishes were corrected using Abbott’s formula (Abbott 1925) before GLM and Tukey’s analysis. Data from pot experiments were analyzed with Student’s *t*-test to compare each treatment and control. Prior to analysis, percentage mortality data from all experiments were arcsine-transformed to normalize variance. Statistical analyses were performed in SPSS version 29 (SPSS 2023).

## Results

### Effects of *Trichoderma* spore suspensions on different biological stages of *Tetranychus urticae*

*Trichoderma* spp. were successfully re-isolated from mite cadavers placed on selective medium, confirming fungal infection in treated individuals. *Trichoderma* spore suspensions, alone or in combination, showed no efficacy against *Tetranychus urticae* eggs. Mortality remained negligible, reaching only 1.5 − 2.8% by 7 dpa. There was no significant statistical difference between the *Trichoderma* treatments and the control at 7 dpa (F = 0.824, df = 10, *P* > 0.05). The data are not shown in figures.

**Fig. 1:**
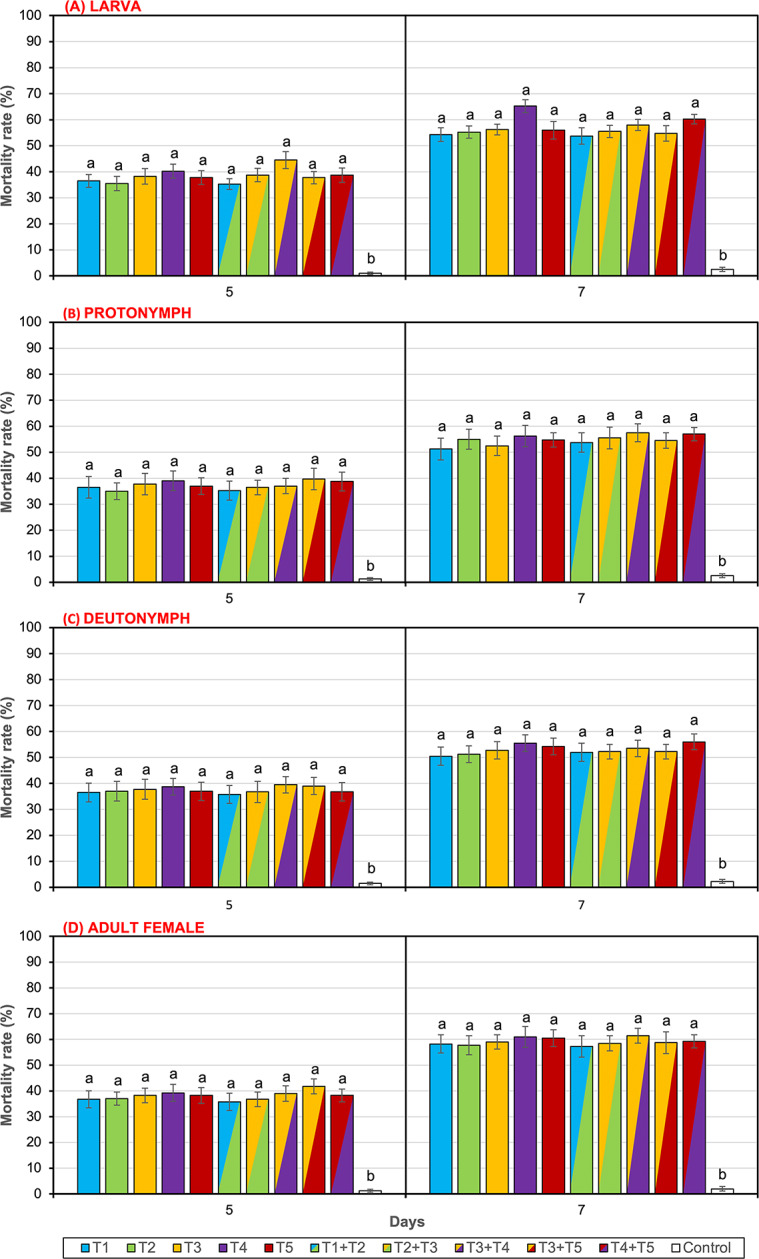
Mortality rates (%) of different biological stages of *Tetranychus urticae* (culture population) after treatment with spore suspensions and their combinations of *Trichoderma* species at 5 and 7 days post-application (dpa) in Petri dish experiment. (**A**) Larva, (**B**) Protonymph, (**C**) Deutonymph, (**D**) Adult female. T1 = *T. guizhouense* Tr49, T2 = *T. guizhouense* Tr118, T3 = *T. harzianum*, T4 = *T. afroharzianum*, T5 = *T. virens.* Different letters above bars indicate significant differences among treatments. Data are presented as mean ± SE

Significant mortality was observed in all mobile stages (larva, protonymph, deutonymph, and adult female), with mortality increasing over time and peaking at 7 dpa. At 5 and 7 dpa, mortality ranged from 35.3 to 44.5% and 53.8–65.3% in larvae, 35.0–39.8% and 51.3–57.5% in protonymphs, 35.8–39.5% and 50.5–56.0% in deutonymphs, and 35.8–41.8% and 57.3–61.5% in adult females, respectively. Significant differences were detected between *Trichoderma* treatments and the control at both 5 dpa (larva: F = 29.281; protonymph: F = 9.085; deutonymph: F = 9.554; adult female: F = 13.671; df = 10, *P* < 0.001 for all tests) and 7 dpa (larva: F = 35.639; protonymph: F = 17.070; deutonymph: F = 20.548; adult female: F = 19.896; df = 10, *P* < 0.001 for all tests). However, no significant differences were observed among the *Trichoderma* species (*P* > 0.05; Fig. 1A, B, C, and D).

### Effects of spore suspension of *Trichoderma afroharzianum* on different spider mite species and populations

#### Petri dish experiments

**Fig. 2:**
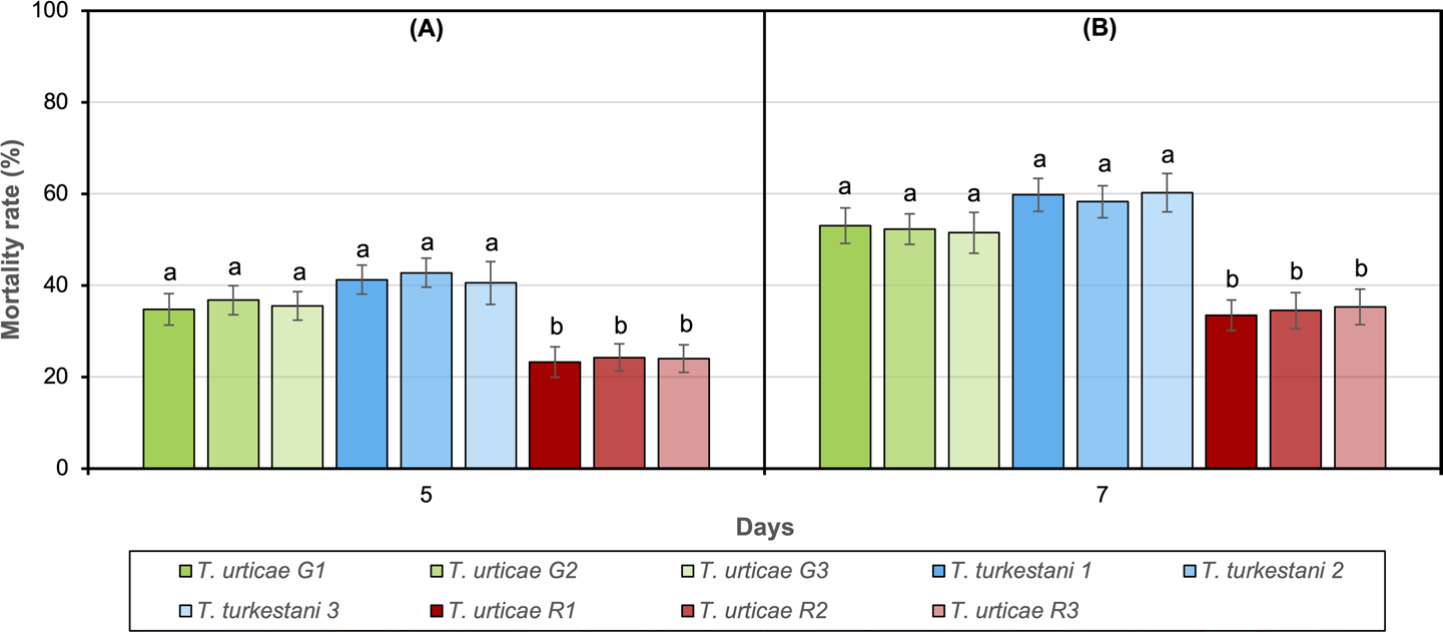
Mortality rates (%) of adult females of *Tetranychus turkestani* and *T. urticae* (green and red forms) after treatment with *Trichoderma afroharzianum* spore suspension in Petri dish experiments. (**A**) 5 days post application (dpa), (**B**) 7 dpa. Different letters above bars indicate significant differences among treatments. Data are presented as mean ± SE. G1–G3: green form of *T. urticae*; R1–R3: red form of *T. urticae*; 1–3: *T. turkestani* populations (see Table 1 for population details)

Spore suspensions of *Trichoderma afroharzianum* at 5 dpa and 7 dpa resulted in the highest mortality in adult females of *Tetranychus turkestani* (40.5–42.5% and 58.3–60.3%) and the green form of *T. urticae* (34.8–36.8% and 51.5–53.0%), whereas significantly lower mortality was observed in the red form of *T. urticae* (23.3–24.3% and 33.5–35.3%) (5 dpa: F = 5.119; 7 dpa: F = 7.846; df = 8, *P* < 0.001 for all tests; Fig. 2A, B). The mortality rate was not found to be statistically significant among the populations of each spider mite species or form (*P* > 0.05). Control mortality at 7 dpa ranged from 1.5% to 3.4% across species and populations.

#### Pot experiments

The numbers of viable eggs and live mobile stages of *T. turkestani* for *T. afroharzianum* and the control were 115.5 and 180.3 for eggs, 106.2 and 170.8 for mobile stages at 7 dpa, respectively (Table 2). Significant differences for eggs and mobile stages were observed between *T. afroharzianum* and control (*P* < 0.001). Compared to the control, *T. afroharzianum* caused a 35.9% reduction in eggs and 37.8% in mobile stages of *T. turkestani*. Similarly, *Trichoderma* spore suspension decreased 38.5% and 38.4% for eggs and 35.6% and 38.6% for mobile stages of *T. urticae* (green) and *T. urticae* (culture), respectively. Both egg and mobile stage counts were significantly lower than the control in *T. urticae* (green) and *T. urticae* (green form, laboratory culture population) (*P* < 0.001; Table 2). In contrast, the egg and mobile stage numbers of *T. urticae* (red form) decreased by 8.7% and 16.6%, respectively, following *T. afroharzianum* treatment, but these reductions were not statistically significant for both eggs and mobile stages (*P* > 0.05; Table 2).

**Table 2:** Mean numbers (± SE) of eggs and mobile stages of spider mites at 7 days post-application (dpa) in pot experiments following treatment with *Trichoderma afroharzianum* spore suspension

		*Trichoderma afroharzianum* ^2^	Control	t value	P
*Tetranychus turkestani*	Egg	115.5±4.0 b^3^	180.3±7.1 a	7.971	*P* < 0.001
	Mobile stages^1^	106.2±1.9 b	170.8±6.3 a	9.845	*P* < 0.001
*Tetranychus urticae* (green)	Egg	149.7±5.8 b	243.6±3.7 a	13.609	*P* < 0.001
	Mobile stages	158.8±6.4 b	246.4±5.8 a	10.173	*P* < 0.001
*Tetranychus urticae* (culture)	Egg	162.3±5.8 b	263.5±3.1 a	15.341	*P* < 0.001
	Mobile stages	174.1±4.7 b	283.5±5.5 a	15.050	*P* < 0.001
*Tetranychus urticae* (red)	Egg	229.0±6.2 a	250.7±7.2 a	0.341	*P* > 0.05
	Mobile stages	210.1±8.1 a	252.0±9.3 a	0.726	*P* > 0.05

^1^Total number of mobile stages (larvae, protonymphs, deutonymphs and adults), ^2^The number of live individuals, ^3^Different letters within a row indicate significant differences between the treatment and control

## Discussion

This study evaluated the acaricidal effects of spore suspensions from four *Trichoderma* species against *T. urticae* (green and red forms) and *T. turkestani* populations associated with cotton. The results showed that *Trichoderma* spores were ineffective against mite eggs—an outcome consistent with previous studies, which report higher egg resistance than mobile stages (Chandler et al. 2005; Bugeme et al. 2009). However, significant mortality was recorded across larval, nymphal, and adult female stages across all *Trichoderma* treatments. Efficacy was highly variable among mite species and populations; notably, the red form of *T. urticae* exhibited lower susceptibility in both Petri dish and pot experiments.

The ineffectiveness of spore suspensions against eggs stems from several factors. Primarily, the egg chorion acts as a robust physical barrier that restricts fungal adhesion, germination, and penetration (Dogan et al. 2017). Furthermore, the absence of respiratory openings and a hemocoel —where fungal cells propagate in mobile stages—along with the lack of feeding activity means the eggs offer no pathway or environment suitable for fungal infection (Afifi et al. 2007; Zhang et al. 2014). In contrast, studies reporting significant ovicidal activity often used fungal filtrates or metabolites (Meteab et al. 2022; Evren et al. 2025). This suggests that crude metabolite preparations contain compounds that can diffuse into eggs or disrupt embryonic development, highlighting the need to explore formulations that combine spores with secondary metabolites or adjuvants to enhance ovicidal potential.

In contrast to eggs, mobile stages showed high susceptibility with mortality reaching 50–65% at 7 dpa. These results align with previous studies documenting the use of *Trichoderma* spore suspensions, which reported similar or higher mortality rates for adult and immature mites (e.g., *T. harzianum* at 62–64%, *T. album* at 86% and even higher for *T. longibrachiatum* at ≈ 88%) (Afifi et al. 2007; Elhakim et al. 2020; Awad and Hendawy 2023; Çobanoğlu et al. 2024). Comparable results have been obtained with metabolites such as 6-pentyl-α-pyrone and crude filtrates of *T. afroharzianum* and *T. virens* (Sholla and Kottb 2017; Meteab et al. 2022; Evren et al. 2025). These findings confirm that *Trichoderma* spp. act most effectively on mobile stages, likely through combined effects of cuticle penetration, enzymatic degradation, and toxic metabolites (Hermosa et al. 2014; Zeilinger et al. 2016).

The tested *Trichoderma* isolates differed in their effects on spider mites, and no significant differences were detected among species or their combinations, indicating a lack of synergistic interactions. Similar variability in acaricidal activity among *Trichoderma* species and isolates has been reported previously (Afifi et al. 2007; Sholla and Kottb 2017; Meteab et al. 2022; Awad and Hendawy 2023; Evren et al. 2025). The absence of synergistic effects among combined isolates may be attributed to potential antagonistic or competitive interactions, which can limit the production or activity of bioactive metabolites. Such variability underscores the critical importance of strain-specific traits—including metabolite profiles, enzyme activity, and spore hydrophobicity—which directly influence overall pathogenicity (Vinale et al. 2009). Furthermore, the moderate efficacy observed, particularly for *T. afroharzianum*, suggests that its mode of action is likely multifactorial, involving enzymatic degradation (e.g., chitinases and proteases), secondary metabolites, and interactions with the mite cuticle, rather than rapid acute toxicity.

The differential susceptibility observed among spider mite species and populations is particularly noteworthy. *T. afroharzianum* induced higher mortality in *T. turkestani* (up to 60%) and the green form of *T. urticae* (53%), but its effect was significantly lower in the red form (35%) in Petri dish experiments. Such variation among populations may be related to physiological or biochemical traits such as cuticle thickness, detoxification enzymes, stress tolerance that confer pesticide resistance might also reduce susceptibility to fungal infection by interfering with fungal penetration or metabolite activity (Zhang et al. 2022; Barnes et al. 2024). Therefore, population-specific responses must be considered when developing biocontrol strategies.

Pot experiments in this study confirmed the laboratory results but also highlighted the challenges of achieving high control levels under more realistic conditions. While *T. afroharzianum* reduced egg and mobile stage numbers of *T. turkestani* and green form *T. urticae* by 35–39%, its effect on red form populations was negligible. These values are still below the efficacy typically required for standalone field control. Similar trends have been reported for other entomopathogens: Bugeme et al. (2009) observed lower virulence of *Metarhizium anisopliae* against *T. urticae* under fluctuating temperatures, while Dogan et al. (2017) emphasized the need for combining fungal pathogens with predatory mites to achieve sustainable suppression. Therefore, while *T. afroharzianum* shows promise, its moderate efficacy suggests it is best employed within an integrated pest management (IPM) framework.

Overall, the results of this study align with the growing literature supporting the role of *Trichoderma* spp. as dual-purpose organisms: plant growth-promoting fungi and pest suppressors (Zin and Badaluddin 2020; Poveda 2021; Yao et al. 2023). However, as Pozo et al. (2024) emphasized, their efficacy as biocontrol agents can vary depending on species, environment, and target pest. Our findings indicate that spore suspension of *T. afroharzianum* alone is unlikely to provide reliable suppression of spider mites in cotton, particularly against resistant red form populations of *T. urticae*. Thus, integrating *Trichoderma* with other biological control agents (e.g., predatory mites such as *Phytoseiulus persimilis* or *Neoseiulus californicus*) and selective acaricides may be a more effective strategy (Cakmak et al. 2005, 2009; Tanaka et al. 2023).

In conclusion, this study demonstrates that *Trichoderma* spore suspensions, particularly those of *T. afroharzianum*, have moderate but significant acaricidal effects on mobile stages of spider mites under controlled conditions. The lack of ovicidal activity and reduced efficacy against resistant red form populations highlight the limitations of using *Trichoderma* alone. Nonetheless, their incorporation into IPM programs may contribute to reducing acaricide reliance and managing resistance, although their efficacy should be validated under field conditions and on cotton plants, particularly under varying environmental conditions such as temperature and humidity.

## Data Availability

All datasets generated and analyzed in this study are available from the corresponding author upon reasonable request.
